# Retraction Note: Knowledge, attitude and practices in relation to prevention and control of schistosomiasis infection in Mwea Kirinyaga county, Kenya

**DOI:** 10.1186/s12889-021-10358-y

**Published:** 2021-02-09

**Authors:** J. Mwai, S. Njenga, M. Barasa

**Affiliations:** Kenya Medical Research Institute, Box 54840-00200, Nairobi, Kenya

**Retraction Note: BMC Public Health 16, 819 (2016)**

10.1186/s12889-016-3494-y

The Editor has retracted this article [1] because it contains sections that substantially overlap with the following articles [2–5]. Author J. Mwai agrees to this retraction. Authors S. Njenga and M. Barasa have not responded to any correspondence from the editor or publisher about this retraction.
